# Evidence‐Based Education for Optometrists Working with Patients with Dementia

**DOI:** 10.1002/alz70858_098701

**Published:** 2025-12-25

**Authors:** Marianne Emma Florence Piano, Bao N Nguyen, Ambhruni Padhye, Kwang Cham, Lynette Joubert, Allison M McKendrick

**Affiliations:** ^1^ National Centre for Healthy Ageing, Monash University & Peninsula Health, Melbourne, VIC, Australia; ^2^ National Vision Research Institute, Australian College of Optometry, Melbourne, VIC, Australia; ^3^ Department of Optometry and Vision Sciences, University of Melbourne, Melbourne, VIC, Australia; ^4^ Department of Biomedical Engineering, University of Melbourne, Melbourne, VIC, Australia; ^5^ Department of Social Work, University of Melbourne, Melbourne, VIC, Australia; ^6^ School of Allied Health, University of Western Australia, Perth, Western Australia, Australia; ^7^ Lions Eye Institute, Perth, Western Australia, Australia

## Abstract

**Background:**

Global reports highlight the importance of access to eyecare for older adults living with dementia in communities, to help maintain independence. However, people living with dementia experience barriers to accessing routine eyecare and are less likely to see an optometrist than people without dementia. Optometrists experience challenges assessing people living with dementia, addressable with evidence‐based education/training. This research aimed to: 1) Explore the impact of completing an evidence‐based education intervention upon knowledge, attitudes and practice of optometrists regarding older adults living with dementia in the community. 2) Identify barriers and facilitators impacting translation of learning into changes in practice.

**Method:**

This mixed method evaluation study surveyed knowledge of dementia, attitudes towards people with dementia and current practice in dementia‐friendly eyecare. Surveys were the Dementia Knowledge Assessment Scale (DKAS), Dementia Attitudes Scale (DAS), and Confidence in Dementia Scale (CODES), completed pre‐course, post‐course, and one year post‐course. Also, a practice pattern survey derived from existing clinical practice guidelines was completed pre‐ and one year post‐course. Quantitative survey data was complemented by qualitative semi‐structured interviews with learners completing the course, exploring learning experiences and intentions to apply learning, through the lens of an established behaviour change framework (COM‐B).

**Result:**

38 optometrists participated in the evaluation; 15 have completed the course to date. Short‐term outcomes are reported. Mean changes in knowledge and attitudes about dementia (DKAS and DAS scores) were modest (DKAS: +1.69 ± 0.82, range 0‐3 points; DAS: +1.92 ± 4.37 (range ‐3‐15 points). Confidence working with people with dementia (CODES score) increased by mean 5.46 ± 4.97 points (45 point scale, range ‐2‐15 points). Emerging themes from 7 interviews around barriers and facilitators to translation of learning to practice include needing dementia training for eyecare practice staff, and impacts of retail optometry KPIs upon ability to deliver dementia‐friendly eyecare.

**Conclusion:**

The course attracted considerable interest, demonstrating a role in addressing unmet education/training needs identified from our research. Increasing optometrist capacity to provide dementia‐friendly eyecare through an accessible, online course makes it easier for people living with dementia and family carers to find an optometrist who knows dementia, supporting access to eyecare.

